# Manipulating the reported age in earliest memories in a Dutch community sample

**DOI:** 10.1371/journal.pone.0217436

**Published:** 2019-05-31

**Authors:** Birte Klusmann, Ineke Wessel

**Affiliations:** 1 Department of Health Psychology, Section Health Sciences, University Medical Center Groningen, Groningen, The Netherlands; 2 Department of Clinical Psychology and Experimental Psychopathology, University of Groningen, Groningen, The Netherlands; Middlesex University, UNITED KINGDOM

## Abstract

**Background:**

Childhood amnesia in adults can be defined as the relative paucity of autobiographical memories from the first years of life. An earlier study by Wessel, Schweig and Huntjens demonstrated that ‘how’ we ask for an earliest memory may bias adults’ estimations of when the earliest childhood memory actually happened. They suggested that snapshot memories (i.e., mental pictures) were less sensitive to an age manipulation than event memories (i.e. narratives). We aimed at replicating and extending these findings using a Dutch community sample stratified for age, gender and educational level.

**Method:**

Participants (N = 619) were randomized into one of three experimental conditions. Prior to recalling their earliest memory, participants in the early and late conditions were presented with examples referring to memories from age 1–2 or 5–6, respectively. The example memories in the control group did not contain any age cues. Participants reported the estimated age in their earliest memory and their strategy for arriving at this estimate. They also rated their memory’s phenomenology (e.g. vividness). Independent judges rated memory type (e.g., snapshot memories).

**Results:**

Compared to the control group, participants in the early condition estimated the age in their memory to be significantly earlier. The difference between the late and control conditions was too small to be of interest. We did not observe a statistically significant interaction between memory type and condition. Snapshot memories were from a younger age than event memories and showed differences with respect to phenomenology (e.g., emotional intensity).

**Conclusion:**

The results of this community study replicate earlier findings that instructions including age cues influence estimates of age in earliest memories. Although snapshot and event memories seem to be qualitatively different, the idea that they respond differently to an age manipulation could not be corroborated.

## Introduction

Whereas the majority of our memories come and go, there is a period of life from which we reliably fail to remember much, if anything at all. This phenomenon is known as infantile or childhood amnesia [1]. It is defined as the relative paucity among adults of verbally accessible autobiographical memories from the first 3 to 4 years of their life [2], [3]. Overall results of studies into the reported age of earliest memories in Western cultures converge on a grand average of 3.5 years [4]. However, the reported age in early memories may not be as stable as such a consistent pattern of results suggests.

Several experimental studies looked at variables that might systematically bias the reported age in earliest memories. To begin with, age reports appear to be sensitive to social pressure. In a study by Peterson, Kaasa and Loftus [5], participants overhearing confederates talking about very early experiences reported significantly younger earliest memories than unexposed participants. Furthermore, Malinoski and Lynn [6] subjected their participants to an extensive probing procedure, including suggestion and visualization, and found that the participants reported a mean age of 1.6 years in their memories (as opposed to a mean reported age of 3.7 years in initial memories). Because the reported age brought about by their procedures was well within the childhood amnesia period, the authors of both studies interpreted these memories as improbable. Especially Malinoski and Lynn’s procedure contained strongly suggestive features that have been associated with false memory implantation (e.g. [7], [8]). A recent study [9] even found that 40% of a large sample reported memories from the first two years of life. The authors interpreted these as representing the (inadvertent) construction of fictional mental representations of early childhood situations.

Still, memories with a reported age before age 3 are not necessarily false. There is some evidence to suggest that such early memories are retained and may be even misdated such that they appear to reflect later occurrences [10], [11]. An explanation for misdating is that time estimation in autobiographical memory is complicated by the absence of date information in personal memories. When people are asked to give a date, they presumably base their estimate on variables such as memory characteristics (e.g., clarity; ease of access) or context information provided by other landmark memories, for example their first day at school or moving house [12]–[14].

Consistent with a reconstructive account relying on autobiographical knowledge, experimental procedures capitalizing on the recall of information from a certain age rather than social pressure have been shown to affect the average reported age in earliest memories. For example, Kingo and colleagues [15] showed that retrieving memories from age 3 as a ‘warm up’ exercise prior to recalling earliest memories resulted in younger earliest memories than a ‘warm-up’ exercise involving memories from age 6. In a study by Wessel et al. ([16], study 2), participants answering general knowledge questions that referred to their preschool years (e.g., “Do you know the color of the family car in your preschool years?”) reported younger earliest memories than participants who had solved arithmetic problems. Interestingly, participants who answered questions about general news events (“Did the Berlin wall fall during your preschool years?”) also reported younger ages in their earliest memories.

This latter finding suggests that autobiographical context information may not even be necessary to explain the malleability of age reports in earliest memories. Indeed, other studies have shown that even relatively mild manipulations, such as reading examples of memories from a certain age ([16], study 1), or thinking about whether something happened before or after a certain age [17], influence average age estimates. As estimating the age in earliest memories can be taken as an instance of judging under uncertainty, Greenberg and colleagues proposed that such subtle effects may result from anchoring. The term anchoring was coined by Tversky and Kahneman [18] and refers to the phenomenon that people’s number estimates are biased towards a particular initial value. The idea is that people use a starting point when asked to estimate a number that they are uncertain about. This starting number or anchor may be suggested by the experimental context (e.g., in an instruction or question). Subsequently, people adjust this initial value to arrive at their final estimate, but do so insufficiently, resulting in bias.

Wessel and colleagues tried to disentangle an interpretation in terms of using autobiographical knowledge from anchoring in order to explain their finding that reading vignettes about a young age (e.g. a story about someone’s second birthday) yielded younger reported ages than reading vignettes containing older ages (e.g., a story about someone’s sixth birthday). The participants in this study had answered questions about the strategy they used to arrive at their age estimate. The authors reasoned that if anchoring was important, then age estimations in participants who said to have guessed rather than looked for context in autobiographical knowledge [12] should be particularly sensitive to the age manipulation. However, Wessel et al. ([16], study 1) found that guessers exposed to early age cues reported *later* memories than participants who reported to have used autobiographical knowledge. This contradicts predictions derived from an anchoring account. Yet, it should be noted that this analysis was exploratory and that this finding was not replicated in an exploratory analysis of the results of a second experiment [16].

Further exploratory analyses of the results of Wessel et al. [16]’s first experiment raised the intriguing possibility that the type of memory might be important in yielding varying age estimates. Bruce et al. [19]distinguished between two types of memories. An *event memory* reflects a memory containing a substantial amount of information, that is, a sequence of related events, a narrative structure, a context, and a setting or background. Alternatively, *fragment memories* consist of nothing more than a brief experience, e.g. a sensory impression (visual, auditory, or olfactory), or “a transient feeling or emotion, or a momentary behavior or action” ([19]; p. 308). Interestingly, age estimates of fragment memories have been reported to be significantly younger than those of event memories, and it has been suggested that fragment memories may originate from earlier developmental periods [19], [20]. However, Wessel et al. [16] did not find any fragment memories in their data when using Bruce et al. [21] ‘s rather strict definition of fragments as fleeting sensations or images without any context or autobiographical knowledge (e.g., the image of a patterned quilt). Yet, the memories did seem to vary in terms of level of elaboration, and the authors added a category of snapshot memories. These were defined as mental pictures that may contain some context, but miss the narrative quality of event memories (e.g., the more elaborated description of lying in bed and feeling grandfather patting one on the back, including a description of the location of the bedroom in the house). Wessel and colleagues categorized 45% of the memories in their sample as snapshot memories. Similar to earlier findings for fragment memories [19], they found that overall, the age in snapshot memories was significantly younger than in event memories [16]. In addition, in their first experiment, Wessel et al. [16] observed that the effect of the age manipulation varied with memory type (i.e. snapshot and event memory) such that the reported ages in event memories were significantly higher in the late condition than in the early condition, whereas the reported ages in snapshots did not seem to differ across conditions. Thus, it may be that snapshots are relatively insensitive to the age manipulation. Yet, this finding was not replicated in their second experimental study ([16], study 2). All in all, exploratory analyses of Wessel et al.’s ([16], study 1) data yielded results regarding the use of strategies and memory type that were not corroborated in a second experiment. Perhaps these null-findings were due to substantial differences in the design of the studies. That is, the follow-up study aimed at priming the preschool age-period (0-3- years) by asking knowledge questions. Thus, this study did not use vignettes and, more importantly, did not contain a late age (i.e. 5–6 years) condition. Alternatively, data-dependent decisions in exploratory analyses increase the probability of type I error (see [22]). That would imply that the findings in the initial study may be spurious. Clearly, a sufficiently powered replication of the original study is warranted.

The aim of the present study was to provide a relatively direct replication of Wessel et al.’ first experiment in a large sample. Thus, participants read vignettes referring to events happening at a young (1 or 2 years of age) or relatively late (5 or 6 years) ages, reported their earliest memory and estimated their age in this memory. We adapted the original design in several ways. To begin with, we added a control condition, allowing comparisons of participants receiving age information with participants who were not exposed to any age cues. Furthermore, we used a Dutch community sample, because many studies in the infantile amnesia literature rely on college student samples (e.g. [13], [14], [23], [24]), limiting the generalizability of the findings. We used a sample stratified by age, gender, and level of education following the example of a study in a Danish community sample [25]. The results of this study suggest that variations in the age of first memories may be associated with gender and education. Finally, we limited our sample to native Dutch participants to minimize cultural variability. It has been reported that on average, individuals from Western individualistic cultures report earlier memories than Eastern collectivistic cultures (e.g. [26], [27]). Finally we used a different, shorter measure of autobiographical memory characteristics than the 66-item questionnaire in Wessel et al. [16] because anecdotical reports indicated that participant had found it long and tedious.

In the present study we aimed at replicating the findings reported by Wessel et al. by following their analytic strategy as closely as possible. We expected that 1) compared to the control group, age estimates would be younger in the early condition and older in the late condition; 2) more participants in the early than late condition would report using a guessing strategy to estimate their age; 3) there would be no differences of interest in confidence ratings across conditions; 4) participants who guessed their age would be more sensitive to the age manipulation than participants who used autobiographical knowledge, such that reported ages in guessers would be younger in the early and older in late condition compared to the control group; 5) the early condition would report more snapshot memories than the late condition; 6) condition and memory type would interact such that event memories would be dated as older by participants in the late condition and younger in the early condition, whereas no differences of interest between the conditions would emerge in the estimated age of snapshot memories; 7) snapshot memories would differ from event memories in terms of phenomenology (e.g., valence, vividness, coherence).

## Materials and methods

### Availability of materials and data

All materials, supporting information and the anonymized data of this study are publicly available at https://osf.io/6ekxd/.

### Study design and power analysis

The study had a 3 (instruction: early, late, no-age control) x 2 (memory type: snapshot, event) between-groups design. For allocating participants, we used a randomized block design, equally distributing gender (male / female), education level (low / medium / high) and age (20–29; 30–39; 40–49; 50–59) across the early / late / no-age instruction groups. Memory type was experimenter-rated after data collection. Thus, the number of participants and the distribution of the demographic variables across memory type was not determined in advance.

Wessel et al. ([16], study 1)’s findings in undergraduates indicated that the interaction between instruction and memory type had a small to medium effect size (i.e., η_p_^2^ = .039). An a priori power analysis with *f* = .15, α = .05, and power = .90 indicated that *n* = 95 per cell would be required if each instruction condition would contain equal numbers of snapshot and event memories. However, based on the earlier results we expected to find 40% of the memories to be snapshot memories and, therefore, aimed at recruiting a total *N* = 720 with *n* = 240 in each of the three instruction conditions.

### Recruitment and respondents

A community sample of native Dutch participants was recruited by the market research company PanelInzicht (https://www.panelinzicht.nl/). Initially, 1735 participants were screened for eligibility. Participants were eligible if their ethnicity was Dutch and if they were between 20 and 59 years old. A total of 680 participants were screened out because they failed to meet the inclusion criteria (*n* = 228) or because their stratum was already filled (*n* = 452). A total of 1055 participants were eligible and started the questionnaire. Of those, 327 did not finish it. The data collection continued until the data for 728 completers had been obtained. However, of those, an additional 109 were excluded because they 1) reported that they had not completed the questionnaire truthfully (*n* = 10); 2) did not report a (adequate) memory (*n* = 54, see S1 File for exclusion criteria); 3) reported memories from age 11 or older (*n* = 43; [17]) or 4) reported that they had used an external source (e.g., doctor’s notes) at the time of filling in the questionnaire to look up their age in the memory (*n* = 2). Thus, the final sample consisted of 619 respondents, who were randomly allocated to the three instruction conditions (control: *n =* 212, early: *n =* 204; late: *n =* 203). S1 Table provides a detailed overview of how many participants remained in each of the strata and how instruction conditions were distributed across strata. All participants who completed the full questionnaire received a small monetary reward (i.e. 2.75€). The Ethical Committee Psychology (ECP) of the University of Groningen approved the study.

### Materials and procedure

The market research company PanelInzicht sent invitations to relevant groups of potential participants among registered members of their research panel. Participants received an e-mail and were directed to an online questionnaire that was hosted at the University of Groningen. The questionnaire was constructed with Qualtrics [28]. All questions/ instructions were in the Dutch language and were for a large part direct translations of the English version used by Wessel et al. ([16], study 1). The original questionnaire, including translations into English, can be found at https://osf.io/6ksx3/. The questions were presented in the following order.

#### Informed consent and Screening

First, respondents gave their informed consent. Next, five questions assessed demographic information. Participants typed in their current age in years. Based on their answer they were allocated to one of the four age-categories (20–29; 30–39; 40–49; 50–59). Next, they indicated their gender (female / male) and their educational level choosing one of three options corresponding to high, middle and low levels in the Dutch educational system according to the categorization by the Netherlands National Institute for Public Health and the Environment (i.e., high = Research / Applied Science University (WO / HBO); middle = Highschool / middle vocational education (VWO, HAVO, MBO 2–4); low = Primary school / lower secondary school / lower level vocational education (Basisonderwijs, LBO, MAVO, VMBO, MBO-1, AVO-onderbouw [29]). Ethnicity was assessed by asking the country of birth (the Netherlands / other) for each of the participants’ parents separately. Only if both parents were born in the Netherlands, Dutch ethnicity was assumed [30]. Age and ethnicity were used as inclusion criteria. If one of the criteria was not met, or a stratum had already been filled sufficiently, participants were screened out and directed to the PanelInzicht webpages. Participants fulfilling all inclusion criteria were allocated randomly to one of the three instruction conditions, with the restriction that each condition contained equal numbers of participants from each age, gender, and educational level group.

#### Earliest memory & age manipulation

Next, three examples of earliest memories, containing specific age information, were provided. Respondents in the early condition read Wessel et al.’s examples referring to memories from age 1–2 and those in the late condition saw examples reflecting memories from age 5 to 6. As the original study ([16], study 1) lacked a no-age control group, the examples were adapted to provide versions without any further age information. For all instruction conditions the examples contained an event memory, a fragment memory, and a memory description which included characteristics of both types. Next, participants were instructed to write down their earliest memory. The memory examples can be found in S2 File.

#### Age estimation, confidence and strategies

The questions in the next section were direct translations of those in Wessel et al. [16]. Age in the reported memory was rated using drop-down menus for years and months separately. In addition, participants indicated the amount of confidence in their age estimates, using slider scales (0 = not at all sure; 100 = 100% sure). This was done separately for years and months. In order to assess which strategy a respondent had used, three options were provided (‘I simply knew that I was this age’, ‘I used a specific strategy to derive to the age of the memory’ or ‘It was a guess’). Respondents who indicated to have simply known their age in their memory or to have used a specific strategy in order to derive their age were asked to further explain that strategy.

#### Memory characteristics

Different from Wessel et al. [16], memory characteristics were assessed adapting questions from the Memory Experiences Questionnaire (MEQ; [31] and items provided by Bruce et al. ([19]; see S3 File). The order of the questions was randomized for each participant. Items were scored on 11-point Likert scales, ranging from 0 = *totally disagree* to 10 = *totally agree*. In total, 22 questions covered the following domains: (1) “Sensory details” (3 items; α = .630) assessing sensory and perceptual information such as smell, sound, or touch, (2) “Vividness” (2 items; α = .651), (3) “Place details” (2 items; α = .602), (4) “Time perception” (1 item), (5) “Valence of the memory (2 items; α = .921), (6) “Valence of the event when it happened” (2 items; α = .877), (7) “Emotional intensity of the event when it happened” (1 item), (8) “Memory of general feeling (at the time)” (1 item), (9) “Surrounding happenings” (2 items; α = .759), (10) “Sharing” (2 items; α = .697), (11) “Visual perspective” (2 items; α = .484), (12) “Coherence” (1 item), and (13) “Accessibility” (1 item). The internal consistency of the subscale “Visual Perspective” was too low. Therefore, the two items (seeing through one’s own eyes, reflecting field perspective and seeing through someone else’s eyes, reflecting observer perspective) were considered separately. It should be noted that the values of the observer perspective item had been reversed (i.e., 0 = 10; … 10 = 0) to match the field perspective item. We report the reverse-coded values. The Cronbach’s α were calculated using the scores of the subsample that was included in the analyses (n = 440, see below).

In order to control for careless responding, a control question (‘The sun revolves around the earth’) was randomly presented among the memory characteristic items. This item was also rated on an 11-point Likert scale and participants were expected to select ‘*Totally disagree*’.

#### Fragmentation

As in Wessel et al. ([16], study 1), the general characteristics of fragment and event memories were described. Participants were asked to rate the extent to which their memory was similar to a snapshot or an event. The order of the snapshot and event rating scales was random for each participant. Different from Wessel et al. ([16], study 1), 11-point Likert scales were used, (0 = *totally disagree*– 10 = *totally agree*). The event memory rating was reverse-coded. The internal consistency of the index of fragmentation, i.e. both scores, was too low (Cronbach’s α = .569, [32]). We, therefore, treated the scores separately.

#### Final questions and closing

The final section again was a translation from Wessel et al.’ questionnaire and consisted of three questions. In response to an open-ended question, participants typed in what they thought to be the purpose of the study. In addition, they were asked whether they generally believed that it is possible to have memories from the age of two or earlier (Yes / No). Finally, participants were asked if they had answered the questionnaire sincerely and truthfully (Yes / No). The instructions explicitly stated that their answer to this question would not affect them in any way.

Next, participants were referred to the website of PanelInzicht. For participants who had completed the questionnaire, participation codes were extracted from the database and sent to PanelInzicht, who in turn provided those participants with financial compensation.

### Coding memories

Coders were blind to condition and the self-reported amount of fragmentation. Coding was based on the original coding scheme [16], which contained the following categories: “fragment memory”, “snapshot memory”, “event memory”, “repetitive memory”. For the present purpose two categories, i.e. “autobiographical fact/ general memory/ association” and “not to be coded”, were added. The coding criteria can be found in S4 File. The authors (I.W. and B.K.) coded memory types independently for the first 100 cases. The interrater reliability was good, *k =* .795. Disagreements were resolved by discussion. The remainder of the memories was scored by the second author (I.W.).

### Analyses

The reported age in the memory was calculated by multiplying the number of years by 12 and adding the number of months provided by respondents. Normality was assumed for all continuous variables, except for the memory characteristic variables place details, surrounding happenings and accessibility, which showed highly skewed distributions. Nevertheless, because of the large sample size we report parametric tests of untransformed variables.

For hypotheses stating a clear direction, we used one-sided Welch *t*-tests comparing two independent groups. We adopted Welch t-tests as a default following recommendations of Delacre, Lakens and Leys [33]. For hypotheses stating that there would be no group differences, we tested for equivalence using Lakens’ [34] TOST procedure. This method uses two one-sided Welch *t*-tests to test whether an observed effect is significantly smaller / larger than an upper / lower bound smallest effect size of interest (SESOI). Thus, an observed ES that is significantly within the range specified by these upper and lower bounds is interpreted as the absence of the effect. For the current comparisons, we calculated the effect size that Wessel et al. [16], study 1) could have detected with a power of 0.33 (see [34], [35]). Accordingly, the equivalence bounds were set to Cohen’s *d* = -0.26 and *d* = 0.26. We also used the TOST procedure to further specify nonsignificant findings from testing directional hypotheses.

For analyses involving more than one factor we used Analyses of Variance (ANOVAs). Like in Wessel et al. [16], a nominal covariate (i.e., belief in memories younger than age 2) was included as a fixed factor, using custom modeling. In addition, the sequential Holm-Bonferroni procedure was used in order to control for family-wise error rates [36]. Where variables were categorical (e.g., memory types), Chi-square tests were applied.

As for exploring differences in the phenomenology of snapshot and event memories, the check for careless responding (“the sun revolves around the earth”) rendered incorrect responses in 43% of the participants (i.e., they failed to choose “completely disagree”). In retrospect, we believe that this check may have been suboptimal for the current purpose. Because the correct answer would require a negation, the statement would require more close reading and more thinking than the items by which it was surrounded (i.e., judging the extent to which a certain characteristic was present). Therefore, we report two sets of Welch *t*-tests: one including all participants whose memories were coded as a snapshot or an event (*n* = 440) and one for only those participants who had also answered the careless responding statement correctly (*n* = 251). Per set, we employed the Benjamini-Hochberg procedure controlling for False Discovery Rate [36].

## Results

### Age comparisons

Table 1 displays means and standard deviations of reported ages in the early, late and control condition. To test the hypothesis that the age manipulation would affect the reported age in earliest memories relative to the no-age control condition, we conducted two one-sided *t*-tests with a Holm-Bonferroni adjusted *α* to control for type I error (*α* = .025, see [36]). The mean age in the early instruction condition showed a statistically significant difference compared to the no-age control condition, *t*_*Welch*_(413.9) = 3.36, *p* = .0008, *d* = 0.33. The comparison between the late condition and the no-age control was not statistically significant, *t*_*Welch*_(405.5) = -0.37, *p* = .713, *d* = 0.036. We used the TOST procedure to specify this null-finding further. The observed ES (Cohen’s *d* = -0.04) fell significantly within the equivalence bounds (-0.26–0.26), *t*_*Welch*_(405.45) = 2.28, *p* = .012. Taken together, whereas participants in the early instruction condition reported significantly younger ages than those in the no-age control condition, the reported ages in the late condition were comparable to those in the control condition.

**Table 1:** Estimated ages, memory types, fragmentation, strategies, confidence and belief in the possibility in memories from before age 2 across conditions.

		Early (*n* = 204)	Late (*n* = 203)	Control (*n* = 212)
**Age in Memory, months** *M* (*SD*)		47.7 (21.9)	56.0 (25.4)	55.1 (23.2)
**Memory type**				
	Fragment *n (%)*	10 (4.9)	10 (4.9)	8 (3.8)
	Snapshot *n (%)*	79 (38.7)	70 (34.5)	81 (37.7)
	Event *n (%)*	68 (33.3)	72 (35.5)	70 (33.0)
	Repetitive *n (%)*	20 (9.8)	25 (12.3)	25 (11.8)
	General/Autobiographical/ Association* n (%)*	27 (13.2)	26 (12.8)	28 (13.2)
**Fragmentation** *M* (*SD*) Composite score^1^		5.04 (2.43)	4.76 (2.65)	4.77 (2.49)
	Like a fragment	6.07 (2.87)	5.69 (3.33)	5.72 (3.19)
	Like a story	6.00 (2.93)	6.17 (2.91)	6.18 (2.86)
**Way of estimating years** *n (%)*				
	Used a strategy	69 (33.8)	52 (25.6)	71 (33.5)
	Just knew	91 (44.6)	96 (47.3)	100 (47.2)
	Wild guess	44 (21.6)	55 (27.1)	41 (19.3)
**Way of estimating months** *n (%)*				
	Used a strategy	37 (18.1)	40 (19.7)	40 (18.4)
	Just knew	44 (21.6)	44 (21.7)	39 (18.9)
	Wild guess	123 (60.3)	119 (58.6)	133 (62.7)
**Confidence** *M (SD)*				
	Years	74.3 (27.6)	70.7 (30.8)	70.2 (32.1)
	Months	44.1 (37.3)	47.5 (38.6)	42.23 (36.8)
**Belief in earliest memories < 2 y** (%)		98 (48.0)	78 (38.4)	102 (48.1)

^1^ The composite score is the mean of the “like a fragment” and the “like a story” ratings. The “like a story” ratings were reverse-coded prior to averaging.

### Strategies

Table 1 displays the percentages of used strategies per condition. In Wessel et al. ([16], study 1), a statistically significant higher percentage of participants in the early condition reported that they had guessed their age compared to the late condition. Separate tests of the other answer options (i.e. just knowing and using a strategy) had not yielded statistically significant differences between the early and late conditions. In order to directly replicate these findings, we compared the percentages of participants that chose each answer option in the early and late conditions separately. We also explored differences between the early and control conditions. Because these analyses would yield a total of six separate *Χ*^*2*^*-* analyses, we used a Holm-Bonferroni procedure to correct for type I error inflation. The analyses did not yield statistically significant results, neither for the comparisons between the Early and Late conditions (i.e., Guess: *Χ*^*2*^(1) = 1.687, *p* = .194; Specific strategy: *Χ*^*2*^(1) = 3.281, *p* = .070; Just knew: *Χ*^*2*^(1) = 0.295, *p* = .587), nor for the comparisons of the Early and Control conditions (Guess: *Χ*^*2*^(1) = 0.381, *p* = .573; Specific strategy: *Χ*^*2*^(1) = 0.005, *p* = .943; Just knew: *Χ*^*2*^(1) = 0.275, *p* = .600). Thus, unlike Wessel et al. ([16], study 1)’s findings, the present results are inconsistent with the idea that one strategy (i.e., guessing) was used particularly often in one of the conditions. The same was true for the reports of strategy use for coming up with the number of months (Early vs. late conditions: Guess: *Χ*^*2*^(1) = 0.118, *p* = .731; Specific strategy: *Χ*^*2*^(1) = 0.163, *p* = .686; Just knew: *Χ*^*2*^(1) = 0.001, *p* = .979. Early vs Control conditions Guess: *Χ*^*2*^(1) = 0.262, *p* = .609; Strategy: *Χ*^*2*^(1) = 0.005, *p* = .946; Just knew: *Χ*^*2*^(1) = 0.471, *p* = .493).

### Confidence ratings

In order to test the hypothesis that confidence ratings of years and months would not differ between conditions, we employed the TOST procedure for comparisons between each pair of conditions. For the ratings of confidence in the number of years, the observed effect sizes of the difference between the early and control condition (*d* = 0.14) as well as the difference between the early and late condition (*d* = 0.12) did not fall significantly between the equivalence bounds, *t*_*Welch*_(409.1) = -1.25; *p* = .105, and *t*_*Welch*_(400.0) = -1.4, *p* = .082, respectively. For the comparison between the late and the control condition, the TOST procedure showed that the effect size of .02 significantly fell within the equivalent bounds (*t*_*Welch*_(413) = -2,47, *p* = .007). Thus, the late and the control condition were statistically equivalent, whereas the results of the comparison between the early and the control condition remain inconclusive.

The reversed pattern was observed for confidence ratings in months. The TOST procedure showed that the effect sizes of the difference between the early and control condition (*d* = 0.05; *t*_*Welch*_(412.8) = -2.12, *p* = .017) as well as early and late condition (*d* = -0.09, *t*_*Welch*_(404.42) = 1.73, *p* = .042) fell significantly within the equivalence bounds, and were thus statistically equivalent. The equivalence test comparing confidence in months of participants in the late and control condition showed that the effect size of .14 did not fall significantly within the equivalent bounds (*t*_*Welch*_(409,56) = -1.22, *p* = .111). Thus, this result remains inconclusive.

### Bias/Anchoring

Next, we tested the hypothesis that participants who said they guessed the number of years in their age were more sensitive to the age manipulation than participants who used autobiographical knowledge (AK; i.e., participants who either just knew the age or used a specific strategy).

First, we tested whether the age of memories differed significantly between participants who believed that it is possible to have memories from before age 2 (Believers; *n =* 278) and participants who did not believe in that possibility (Non-believers, *n* = 341). We observed significantly younger age estimates in the group of Believers (*M* = 48.3, *SD* = 26.8) than Non-believers (*M* = 56.7, *SD* = 20.3; *t*_*Welch*_(506.8) = -4.34, *p* < .001; *d* = 0.33). Similar to Wessel et al. [16], we controlled for belief in earliest memories prior to two by including it as a fixed factor in a 3 (condition: early vs late vs control) x 2 (AK-use: yes vs no) ANOVA.

The ANOVA yielded a significant main effect of condition (*F*(2, 612) = 7.09, *p* = .001, *ηp*^*2*^ = .023). Thus, the age in the memory differed across the three conditions also when statistically controlling for belief in memories under age 2 (estimated marginal means: early condition: *m* = 47.64, *SE =* 1.97; late condition: *M* = 57.42, *SE* = 1.84; control condition: *M* = 55.3, *SE* = 2.01). Neither the main effect of AK-use (*F*(1, 612) = 3.06, *p* = .081, *ηp*^*2*^ = .005), nor the condition by AK-use interaction effect (*F*(2, 612) = 2.12, *p* = .121, *ηp*^*2*^ = .007), were statistically significant.

### Memory types across conditions

Table 1 also displays the percentages of each memory type across instruction conditions. Note that in contrast to Wessel et al.’ findings ([16], study 1), we did identify a relatively small percentage of fragment memories (4.5%, *n* = 28). An overall *X*^*2*^-analysis of memory types across conditions was not statistically significant, *X*^*2*^(8) = 1.86, *p* = .985, indicating that the null-hypothesis that memory types were equally distributed across conditions cannot be rejected. In addition, we compared the composite fragmentation scores between conditions, using two one-sided *t*-tests. Participants in the early instruction condition and the no-age control condition did not differ significantly, *t*_*Welch*_(413.9) = -1.12, *p* = .263, *d* = 0.11. We used the TOST procedure to specify this null-finding further. The observed ES (Cohen’s *d* = 0.11) did not fall significantly within the equivalence bounds (-0.26–0.26), *t*_*Welch*_(413.86) = -1.53, *p* = .063. Likewise, no significant difference emerged between the late condition and the no-age control, *t*_*Welch*_(408.42) = .012, *p* = .991, *d* = 0.001. The TOST-procedure indicated that the observed ES was significantly between the equivalence bounds of (-) 0.26, *t*_*Welch*_(407.1) = -2.65, *p* = .004. Because of the poor internal consistency of the composite fragmentation index (Cronbach’s α = .569), we also explored differences between the conditions for each of its components using overall one-way ANOVAs. These analyses did not indicate an overall difference between conditions for the extent to which the memory resembled a fragment, *F*_*Welch*_(2, 408.9) = 1.024, *p* = .36, or an event, *F*_*Welch*_(2, 410.0 = .25, *p* = .779. Taken together, like in Wessel et al. ([16], study 1), the idea that the early condition would contain more fragmented memories was not corroborated in the present study.

### Age estimates per memory type

To replicate Wessel et al. [16]’s findings that the manipulation affects the age estimates of different types of memories differently, we selected a subsample (*n* = 440) only including memories rated as snapshots (*n* = 230) or events (*n* = 210) across all conditions.

As in Wessel et al. ([16], study 1), we included belief that earliest memories before the age of 2 are possible as a fixed factor covariate in a 3 (Condition: Early vs Late vs Control) x 2 (Memory type: Snapshots vs Events) ANOVA. Statistically significant main effects for condition (*F* (2, 433) = 3.39, *p* = .035, *ηp*^*2*^ = .015) and memory type (*F* (1, 433) = 20.57, *p* < .001, *ηp*^*2*^ = .045) were found, indicating that overall, snapshot memories were dated significantly earlier than event memories (based on estimated marginal means: *M*_*Difference*_ = 9.05; The estimated marginal mean for Snapshots was 46.46 months (SE = 1.384; SD = 20.96) and for Event memories it was 55.51 months (SE = 1.449; SD = 20.98). There was no statistically significant Condition by Memory type interaction effect (*F* (2, 433) = 0.091, *p* = .913, *ηp*^*2*^ = .0004).

### Age estimates and demographic variables

The mean reported ages of earliest memories in our study were higher than is typically found in studies on early memories (i.e. 3.5 years, [2], [3]). Perhaps this discrepancy is due to the use of a community sample rather than a typical undergraduate sample. Table 2 presents the mean reported ages across the levels of education, gender and age in the no-age control condition, because this group would resemble the samples within the general literature the most. As can be seen in Table 2, the average ages were higher than 50 months across all levels of age, gender and education. Thus, an unusually high age in one of the strata cannot explain the relatively high overall age. As numerically, the mean ages in Table 2 seemed to point towards some differences with regard to age and educational level, we calculated effect sizes. The effect size for the difference between men and women was *d* = .179; for a low vs middle educational level *d* = .048, and for a high vs middle educational level *d* = .237.

**Table 2:** Means (SDs) and Cell sizes per age-group, gender, and educational level in the control condition.

		Control Condition	
		*n*	*M (SD)*
**Age**			
	20–29	49	52.55 (22.46)
	30–39	51	55.71 (24.03)
	40–49	53	51.70 (23.96)
	50–59	59	59.75 (22.00)
**Gender**			
	Male	98	57.35 (25.43)
	Female	114	53.17 (20.94)
**Educational Level**			
	High	73	52.64 (22.26)
	Middle	73	53.74 (23.98)
	Low	66	59.32 (23.00)

A table presenting all means per condition can be found in the S2 Table. Note that we refrained from running a statistical analysis of the association between demographic variables and reported age in our 2 (gender) x 4 (age-group) x 3 (educational level) x 3 (condition) factorial design because this would render many comparisons across small groups (especially where cases are missing), yielding a high probability of false positives [22], [36]. The S2 Table also contains a table with the mean ages per educational level for the youngest age group, as they would be the most comparable with the typical undergraduate participant in psychology studies. In the control group, the mean for 20–29 participants with high or middle education level was equal to 4.25 years (51.03 months), which is only slightly younger than the grand mean of the control group (55.10 months).

### Differences in memory characteristics between memory types

Table 3 presents the characteristics for snapshot and event memories including the participants who incorrectly answered the careless responding check. We adjusted alpha’s to control for False Discovery Rate using the Benjamini-Hochberg procedure [36]. P-values that are smaller than the adjusted alpha are denoted by an * in the table.

**Table 3:** Mean (SDs) of fragmentation scores and memory characteristic ratings across memory types, including test statistics of the differences between means, for all participants with a snapshot or event memory code (*N* = 440).

		Snapshot (*n =* 230)	Event (*n* = 210)				
		*M (SD)*	*M (SD)*	*t*_*Welch*_ *(df)*	*P*^*1*^	*Adjusted alpha*^1^	*d*
**Fragmentation**							
* Like a event*		5.47 (3.04)	7.29 (2.23)	-7.19 (418.96)	< .000001*	.003125	0.682
* Like a fragment*		6.63 (2.94)	4.84 (3.16)	6.16 (426.94)	< .000001*	.003125	0.586
**Sensory details**		5.22 (2.09)	5.79 (2.19)	-2.78 (430.3)	.006*	.034375	0.266
**Vividness**		6.30 (2.03)	7.06 (1.73)	-4.25 (436.2)	.000027*	.021875	0.403
**Place details**		8.19 (1.83)	8.34 (1.64)	-0.89 (437.9)	.376	.05	0.086
**Time perspective**		4.03 (2.68)	4.59 (2.8)	-2.12 (430.3)	.034*	.0375	0.204
**Valence (memory)**		7.25 (2.96)	6.26 (3.10)	3.44 (429.6)	.000651*	.03	0.327
**Valence (at the time)**		6.86 (2.94)	5.60 (3.22)	4.27 (423.9)	.000024*	.02	0.409
**Emotional intensity**		6.57 (2.08)	7.43 (1.82)	-4.69 (437.4)	.000004*	.013333	0.440
**Memory of general feeling**		6.49 (2.68)	6.95 (2.57)	-1.86 (437.0)	.063	.043333	0.175
**Surrounding happenings**		2.75 (2.69)	3.97 (2.78)	-4.627 (431.1)	.000004*	.013333	0.446
**Sharing**		3.91 (2.36)	4.63 (2.52)	-3.08 (427.6)	.002*	.033333	0.295
**Perspective**							
	Own eyes	6.96 (2.69)	7.85 (2.04)	-3.96 (423.7)	.000088*	.026666	0.372
	Observer	3.90 (3.26)	3.60 (3.06)	0.98 (4367.7)	.327	.05	0.095
**Coherence**		6.54 (2.48)	7.71 (1.87)	-5.562 (422.8)	< .000001*	.003333	0.532
**Accessibility**		7.74 (2.05)	7.97 (1.89)	-1.27 (437.6)	.225	.05	0.117

*p < adjusted alpha in Benjamini-Hochberg procedure (See [36])

^1^ According to the Benjamini-Hochberg procedure, comparing *p* and *α*_*ad*j_ levels is stopped at the first instance of *p* < *α*_*adj*._ For sake of completeness we report all p and α_adj_ values in this table_._

Table 4 reports all outcomes regarding the memory characteristics reported by participants who correctly answered the careless responding check. We again adjusted alphas for false discovery rate [36].

**Table 4:** Means (SDs) of fragmentation and memory characteristic ratings across memory types, including test statistics of the differences between means, for all participants with a snapshot or event memory code with a correct answer on the check for careless responding (*n* = 251).

		Snapshot (*n =* 139)	Event (*n* = 112)				
		*M (SD)*	*M (SD)*	*t*_*Welch*_ *(df)*	*P*^1^	*Adjusted alpha*^1^	*d*
**Fragmentation**							
* Like a event*		5.02 (3.17)	7.34 (2.43)	-6.55 (248.43)	.00001*	.009375	0.826
* Like a fragment*		6.70 (3.04)	4.84 (3.28)	4.61 (229.47)	.000007*	.00625	0.588
**Sensory details**		5.03 (2.15)	5.46 (2.21)	-1.55 (234.82)	.123	.03125	0.197
**Vividness**		6.15 (2.01)	6.83 (1.81)	-2.83 (246.08)	.005*	.01875	0.356
**Place details**		8.15 (1.83)	8.43 (1.69)	-1.23 (244.52)	.22	.04375	0.159
**Time perspective**		3.94 (2.67)	4.28 (2.95)	-.951 (226.27)	.342	.046875	0.121
**Valence (memory)**		7.20 (3.03)	6.46 (2.99)	1.94 (239.1)	.053	.028125	0.246
**Valence (at the time)**		6.65 (3.12)	5.64 (3.30)	2.46 (231.69)	.015*	.025	0.315
**Emotional intensity**		6.42 (2.14)	7.51 (1.85)	-4.30 (247.73)	.000024*	.0125	0.545
**Memory of general feeling**		6.34 (2.87)	6.88 (2.70)	-1.55 (242.91)	.123	.03125	0.194
**Surrounding happenings**		2.29 (2.51)	3.27 (2.87)	-2.83 (222.11)	.005*	.01875	0.363
**Sharing**		3.74 (2.36)	4.19 (2.67)	-1.38 (223.19)	.171	.01875	0.179
**Perspective**							
	Own eyes	6.77 (2.89)	7.89 (2.02)	-3.62 (244.42)	.0004*	.015625	0.449
	Observer	3.69 (3.17)	3.13 (3.06)	1.43 (241.17)	.153	.0375	0.180
**Coherence**		6.06 (2.63)	7.46 (2.12)	-4.67 (249.00)	.000005*	.003125	0.586
**Accessibility**		7.79 (1.92)	7.98 (1.89)	-.789 (238.94)	.431	.05	0.100

*p < adjusted alpha in Benjamini-Hochberg procedure (See [34])

^1^According to the Benjamini-Hochberg procedure, comparing *p* and *α*_*ad*j_ levels is stopped at the first instance of *p* < *α*_*adj*._ For sake of completeness we report all p and α_adj_ values in this table_._

## Discussion

The present study was a replication of the first study conducted by Wessel et al. [16]. In line with expectations, participants who read memory examples including ages of 1–2 years dated their memories earlier than control participants who had read examples without age information. The effect size of this difference was in the small to medium range. In contrast to our hypothesis, however, the estimated ages in the control condition did not differ meaningfully from those reported by participants who had read memory examples mentioning ages of 5–6 years. Note that Wessel et al.’s first study did not include a control group. Our observations suggest that the instruction manipulation in their study might have induced a decrease of the estimated age in the early condition rather than an increase in the late condition. It should be noted that control groups were also absent in other studies contrasting early and late conditions. Those studies employed warm-up retrieval [15] or an anchor question [17]. Future studies employing an age-neutral control condition might determine if the effect is more prominent for the early than late condition in these particular procedures as well.

In general, the present findings add to the literature showing that including age information in experimental instructions affects the reported age in earliest memories. In the hopes of shedding more light on the mechanisms underlying such findings, we repeated the exploratory analyses in Wessel et al. [16]’s first study. The first set of analyses focused on participants’ self-reported strategy for estimating the age in their earliest memory. This was pertinent to an anchoring account of reported age manipulation effects [17]. According to such an account, reported ages would be biased towards the age in an experimental instruction because it would serve as a starting point for reaching an estimate. Anchoring is assumed to be most powerful in judgments under uncertainty [18] and therefore Wessel et al. reasoned that the effects of the age manipulation should be most prominent in participants guessing their age. However, our results showed that there were no meaningful differences among the conditions in the percentage of participants who had guessed their age in their memory. In addition, confidence ratings were statistically equivalent across conditions and were generally high (70 to 74% for the year estimates). Most important for the anchoring account, there was no indication that the age manipulation was more effective in people who guessed their age than in people who reported to have reconstructed their age using autobiographical knowledge. These results are in contrast to those of Wessel and colleagues [16], who argued against an anchoring account because in their study the age estimates of guessers in the early condition were older (rather than younger) than those of autobiographical knowledge users. It should be noted that even though the present null results do not support the anchoring account, they do not refute it either. There may be other reasons for the absence of stronger effects in guessers. For example, our strategy measure may have been suboptimal for classifying participants. Alternatively, the age manipulation may affect the reported ages in autobiographical knowledge users for other reasons than anchoring.

The second set of exploratory analyses in Wessel et al.’s first study focused on memory type, inspired by the idea that fragment and snapshot memories might represent experiences from an earlier developmental stage than event memories that have a more narrative character[19]. Perhaps an early age cue causes respondents to search in an earlier life time period than a late age cue. If so, that would result in differences in memory types and age estimates across conditions. In contrast to Wessel et al. [16], we did find a small percentage of fragment memories (about 3–5% per condition). However, we did not observe more fragment and snapshot memories in the early condition than in both other conditions. Following Wessel et al.[16], we solely focused on snapshot and event memories in the remainder of the analyses. Consistent with previous findings [16], [19], we observed that snapshot memories were dated significantly younger than event memories. However, contrary to Wessel et al. [16]’s first study, we did not find that the age manipulation affected age estimates differently for snapshots and event memories. Together with the results of their second study, the present finding represents a second failure to replicate the significant interaction effect between condition and memory type on age estimates reported by Wessel and colleagues[16]. This casts doubts on the interpretation that especially event memories are more sensitive to experimental instructions than snapshots. On a more general note, our study highlights the importance of replication studies in order to understand the validity of effects that have been observed in previous exploratory studies.

The third set of exploratory analyses focused on comparing the phenomenological characteristics of snapshot and event memories in detail. Wessel et al. ([16], study 1), had only observed a few statistically significant differences, that is, that snapshot memories were rated as more fragmented and less often shared than event memories. Overall, the effect sizes of the differences in that study were rather small, and the sample may not have been large enough to detect small effects. The larger sample size in the present study yielded more power to detect small differences. On average, snapshot memories were rated as including fewer sensory details and surrounding happenings, were experienced as less vivid, shorter in duration, less coherent and less often shared than reported event memories. Snapshots were on average experienced with lower emotional intensity and less often seen through one’s own eyes than event memories. Further, the memory as well as the situation as it happened at the time were rated as more positive in case of snapshots. These results replicate previous findings that the characteristics of fragment and event memories differ on multiple characteristics [19]. It should be noted, however, that again, overall the present effect sizes were rather small. Indeed, when we restricted the analyses to only those participants who had passed our rather stringent check for carelessness, fewer statistically significant differences emerged than in the unselected sample. In comparison to event memories, snapshot memories were rated as less vivid, emotionally intense, including fewer surrounding happenings, as less coherent, less often seen through someone’s own eyes and as referring to a more positive situation. Thus, all in all, there seem to be some small qualitative differences between the memory types. This, in combination with the finding that snapshots were dated on average 9 months earlier than event memories may be taken as supporting the idea that snapshots originate from an earlier developmental stage [19].

Overall, it should be noted that compared to the literature on ages in earliest memories, our results show higher average ages across all conditions. For example, the general literature suggests a grand mean of 3.5 years (i.e., 42 months) [4], yet the reported age in the memories of the control group in our study was about a year higher (i.e., 55.1 months). Regarding the age manipulation, the current age estimates of the early (47.7 months) as well as the late condition (56 months) also were older than those in Wessel et al.’s [16] first study (i.e., 38.6 and 47.9 months, for early and late conditions, respectively). The question arises whether the nature of our sample (i.e., a stratified community sample) may have played a role in these discrepancies. Indeed, the mean age in a Danish community sample was also relatively high (i.e., 4.2 years; appr. 50 months). The authors reported that overall, earlier memories were observed in women than in men, and in the more highly than lower educated participants. We refrained from additional statistical testing as these tests were not planned. The results of a subset of comparisons (i.e., main effects of age group, gender and education) within a large array of potential comparisons would be difficult to interpret (see for example [22]). Inspection of the average ages in the no-age control subsamples across age groups, gender or educational level in the present study rendered no clear indications that particular groups systematically reported different ages than other groups. Yet, in line with previous research [25], numerically the data seem to hint towards possible differences with regard to gender (i.e., older in males) and educational level (i.e., older in lower education). It should be noted that the effect sizes of these differences can all interpreted as small (d < .237). Future studies may systematically address the question of whether the reported age in earliest memories differs as a function of demographics. This could be achieved with preregistered data analysis plans and study design which specifically address this question. For now, we see no immediate specific explanation for the relatively high average reported ages in earliest memories in the current sample.

There are a number of methodological issues that warrant attention. The first issue pertains to generalizability. We used a community-based sample with age, gender and educational level as the stratifying variables. However, several reports had to be excluded from our analyses due to different reasons, for example providing an invalid memory. As a consequence, not all strata were completely filled. For example, men between 30 and 39 years with middle or low educational level were underrepresented within the late condition. Relatedly, in order to exclude cultural influences we only sampled native Dutch participants, that is, both parents of the respondent had to be born in the Netherlands. Thus, the results cannot be generalized outside the Dutch culture. Future studies may shed light on whether the findings apply to other cultures as well.

Another methodological issue is that our participants completed the questionnaire online. Although this procedure replicates the method employed by Wessel et al. [16], it has disadvantages. To begin with, people may have used external means (e.g., photos, asking family members) to come up with their age. We looked at the strategy descriptions provided by the participants and excluded those who were explicit about using other sources. Yet, it may be that participants did not reveal this in the questionnaire. Testing participants in a laboratory would prevent such a strategy altogether. Furthermore, participants may have engaged in careless responding. As for the earliest memory, we exclude participants who failed to provide a description or who provided a non-sensical response (e.g., *don’t know* responses, randomly typed letters (*dfsgskdn*). As for the ratings of memory characteristics, we used a control question. However, we observed that a substantial number of respondents did not answer this question as intended. This may of course indicate that about 40% of the responses on memory characteristics were not reliable. Alternatively, in retrospect we believe that this question (“the sun revolves around the earth”) may have been too complex in comparison to the other rating scales because it tapped general knowledge, and the correct answer involved a negation. In addition, it should be noted with regard to the measurement of memory characteristics that the internal consistency of the “fragmentation” and “visual perspective” scales was unacceptably low [32], leading us to consider their individual items separately. Furthermore, the internal consistency of the subscales “sensory details”, “vividness” and “place details” may be interpreted as questionable (Cronbach’s alpha’s between 0.6 and 0.7, [32]). Future studies on characteristics should use a simpler question as catch trial, and may opt for more optimal measures altogether.

In summary, the current replication study adds to the evidence that age estimates of earliest childhood memories can be influenced by preceding age information. This pertained to early rather than to late ages. As for mechanisms, we did not find evidence against an anchoring account. In addition, we did not replicate the earlier finding that the age instructions affect snapshot and event memories differently. Compared to event memories, snapshots showed qualitative differences and were of a younger reported age. It should be noted that overall, the effect sizes were of a small magnitude. Future research may further elaborate on the mechanisms underlying the effects of age information on the reported age of earliest memories. On a more general note, our failure to replicate the results from exploratory analyses in Wessel et al.’s study highlights the importance of adequately powered replication studies to shed light on the validity of such findings.

## Supporting information

S1 File Exclusion criteria for memories. (PDF)

S2 File Memory examples for early, late and control conditions. (PDF)

S3 File Rating scale used to assess memory characteristics. (PDF)

S4 File Coding criteria memory types. (PDF)

S1 Table Distribution of participants across strata. (PDF)

S2 Table Tables of ages estimates per strata. (PDF)
