# Supplementary material for: Manipulating the reported age in earliest memories in a Dutch community sample
Source: PLoS One. 2019 May 31;14(5):e0217436. doi: 10.1371/journal.pone.0217436 (PMC6544230; doi:10.1371/journal.pone.0217436)
Supplement: S1 File — (PDF) [file pone.0217436.s001.pdf]

S1 File

Exclusion criteria for memories

Exclude when memory consisted of

- *'Weet niet' ('Don't know')*
- *'Geen herinneringen' ('No memories')*
- *'Kan nergens opkomen' ('Can't think of something')*
- *Nonsense responses (kjhfakjshf)*

Exclude when memory was obviously not childhood memories, i.e.

- memories from a recent event (e.g., how I met my girlfriend (at age ….)
