# Supplementary material for: Manipulating the reported age in earliest memories in a Dutch community sample
Source: PLoS One. 2019 May 31;14(5):e0217436. doi: 10.1371/journal.pone.0217436 (PMC6544230; doi:10.1371/journal.pone.0217436)
Supplement: S2 File — (PDF) [file pone.0217436.s002.pdf]

S2 File

Memory examples for the early, late and control conditions

(*Dutch Version followed by the English Translation*)

EARLY

<u>*Voorbeeld 1*</u>

Ik herinner me dat ik aan het hoofd van een grote tafel zat. Op schoot bij mijn vader, denk ik. De tafel stond vol snoep en mijn lievelingstaart: aardbeiensmaak en veel slagroom. Er stond een klein Mickey Mouse poppetje bovenop. Mijn hele familie was er, mijn zussen, mijn grootouders, mijn tantes, ooms, neven en nichten. We zaten buiten en de zon scheen. Het moet mijn tweede verjaardag zijn geweest, want ik herinner me dat er twee in het midden van de taart stonden. Ze waren blauw. Iedereen was aan het zingen. Toen glimlachte mijn moeder naar mij en mijn vader tilde me over de tafel zodat mijn hoofd recht boven de taart zou zijn. Ik blies naar de kaarsjes en iedereen blies mee. Iedereen juichte en klapte. Naderhand kwam één van mijn zussen naar mij toe en gaf me een knuffel en een kus. Ik herinner me dat het een blije en fijne verjaardag was. Ik ben nog steeds gek op die taart.

<u>Example 1 (English translation)</u>

I remember myself sitting at the end of a large table. On my father's lap, I think. The table was filled with sweets and they had my favorite cake, strawberry flavor and lots of cream. It had a little Mickey Mouse on top. Everyone from my closest family was there, my sisters, my grandparents, my aunts, uncles and cousins. We were sitting outside and the sun was shining. It must have been my second birthday, because I remember

two little candles being stuck in the middle of the cake. I think, I remember them to be blue. Everybody was singing. Then my mum smiled at me and my dad lifted me up over the table so my head would be right over the cake. I blew out the candles and everybody helped. Everybody cheered and clapped. Afterwards one of my sisters came to hug me and gave me a kiss. I remember it to be a joyful and happy birthday. I still love that cake.

Voorbeeld 2

In mijn eerste herinnering grijp ik iemands vingers met beide handen vast.... Ik hou me aan iemand vast, maar ik weet niet wiens vingers het zijn... Ik voel dat mijn voeten op de grond bonzen, één voet voor de andere. Boem, boem, boem. Ik vind het moeilijk om recht te blijven, mijn lijf wil alsmaar naar één kant omvallen. Ik zie stoeptegels en het gras van een grasveld naast de stoep. Ik was waarschijnlijk één jaar oud, of wanneer kinderen ook maar hun eerste stappen zetten.

Example 2 (English translation)

My first memory is about grasping someone's fingers with both hands…I am holding on to someone, but I can't remember whose fingers they were… I feel the harshness of steps running through my legs. Boom, boom, boom. I have difficulties walking straight, somehow my body wants to bend to one side. I see the tiles of a pavement and the grass of a lawn next to it. I was probably around one years of age, or whenever children start to make their first steps.

Voorbeeld 3

In mijn vroegste herinnering zat ik op de grond. Op zo'n kleed waar een hele stad op getekend is, met straten en gebouwen. Mijn zus zat naast me. Ik denk dat we met Lego aan het spelen waren. Dat vonden we leuk toen we klein waren. Ik speelde eigenlijk best vaak met mijn zus als kind. Ik

kan me niet echt herinneren wat er daarna gebeurde ... We vechten, maar ik weet niet zeker waarom. Ze slaat me met een stuk van de Lego. Ik huil .. ik word boos. Ik krab haar .. En toen kwam mijn moeder binnen. Dat is alles wat ik me herinner. Ik denk dat ik ongeveer twee jaar oud was.

<u>Example 3 (English translation)</u>

In my earliest memory I was sitting on the floor. On one of those carpets that had an entire city drawn on it, with streets, and buildings. My sister was sitting next to me. I think we played with Lego. We loved to do that when we were younger. I actually played a lot with my sister when we were young. I can't really remember what happened next… We fight, but I am not sure why. She hits me with a piece of Lego. I cry.. I get angry. I scratch her.. And then my mum came in. That's all I remember. I think that I was about two years old.

LATE

<u>*Voorbeeld 1*</u>

Ik herinner me dat ik aan het hoofd van een grote tafel zat. Naast mijn vader, denk ik. De tafel stond vol snoep en mijn lievelingstaart: aardbeiensmaak en veel slagroom. Er stond een klein Mickey Mouse poppetje bovenop. Mijn hele familie was er, mijn zussen, mijn grootouders, mijn tantes, ooms, neven en nichten. We zaten buiten en de zon scheen. Het moet mijn zesde verjaardag zijn geweest, want ik herinner me dat er zes kaarsjes in het midden van de taart stonden. Ze waren blauw. Iedereen was aan het zingen. Toen glimlachte mijn moeder naar mij en mijn vader zei dat ik over de tafel heen moest buigen zodat mijn hoofd precies boven de taart zou komen. Ik blies helemaal zelf alle kaarsjes uit. Iedereen juichte en klapte. Naderhand kwam één van mijn zussen

naar mij toe en gaf me een knuffel en een kus. Ik herinner me dat het een blije en fijne verjaardag was. Ik ben nog steeds gek op die taart.

<u>Example 1 (English translation)</u>

I remember myself sitting at the end of a large table. Next to my father I think. The table was filled with sweets and they had my favorite cake, strawberry flavor and lots of cream. It had a little Mickey Mouse on top. Everyone from my closest family was there, my sisters, my grandparents, my aunts, uncles and cousins. We were sitting outside and the sun was shining. It must have been my sixth birthday, because I remember six little candles being stuck in the middle of the cake. I think, I remember them to be blue. Everybody was singing. Then my mum smiled at me and my dad said I should bend over the table so my head would be right over the cake. I blew out the candles all by myself. Everybody cheered and clapped. Afterwards one of my sisters came to hug me and gave me a kiss. I remember it to be a joyful and happy birthday. I still love that cake.

<u>*Voorbeeld 2*</u>

In mijn eerste herinnering grijp ik het stuur van mijn fiets met beide handen stevig vast. Iemand houdt mij vast aan de achterkant van mijn zadel, maar ik kan me niet herinneren wie dat was. Ik voel dat mijn benen rond gaan, ze bewegen de pedalen rond. Zoef, zoef, zoef. Ik vind het moeilijk om recht te blijven, de fiets wil alsmaar naar één kant omvallen. Ik zie stoeptegels en het gras van een grasveld naast de stoep. Ik was waarschijnlijk vijf jaar oud, of wanneer kinderen ook maar leren fietsen.

<u>Example 2 (English translation)</u>

My first memory is about grasping my bike's steering wheel with both hands. Someone is holding on to the back of the saddle, but I can't remember who it was. I feel my legs making circles, moving the pedals around. Whoosh, whoosh, whoosh. I have difficulties keeping a straight line, somehow the bike wants to bend to one side. I see the tiles of a pavement and the grass of a lawn next to it. I was probably around five years of age, or whenever children start to learn riding their bike.

<u>*Voorbeeld 3*</u>

In mijn vroegste herinnering zat ik op de grond. Op zo'n kleed waar een hele stad op getekend is, met straten en gebouwen. Mijn zus zat naast me. Ik denk dat we met Lego aan het spelen waren. Dat vonden we leuk toen we klein waren. Ik speelde eigenlijk best vaak met mijn zus als kind. Ik kan me niet echt herinneren wat er daarna gebeurde ... We vechten, maar ik weet niet zeker waarom. Ze slaat me met een stuk van de Lego. Ik huil .. ik word boos. Ik krab haar .. En toen kwam mijn moeder binnen. Dat is alles wat ik me herinner. Ik denk dat ik ongeveer zes jaar oud was.

<u>Example 3 (English translation)</u>

In my earliest memory I was sitting on the floor. On one of those carpets that had an entire city drawn on it, with streets, and buildings. My sister was sitting next to me. I think we played with Lego. We loved to do that when we were younger. I actually played a lot with my sister when we were young. I can't really remember what happened next… We fight, but I am not sure why. She hits me with a piece of Lego. I cry.. I get angry. I scratch her.. And then my mum came in. That's all I remember. I think that I was about six years old.

CONTROL

<u>*Voorbeeld 1*</u>

Ik herinner me dat ik aan het hoofd van een grote tafel zat. Ik zat op een stoel naast mijn vader, denk ik. De tafel stond vol snoep en mijn lievelingstaart: aardbeiensmaak en veel slagroom. Er stond een klein Mickey Mouse poppetje bovenop. Mijn hele familie was er, mijn zussen, mijn grootouders, mijn tantes, ooms, neven en nichten. We zaten buiten en de zon scheen. Het moet mijn verjaardag zijn geweest, want ik herinner me dat er kaarsjes in het midden van de taart stonden. Ze waren blauw. Iedereen was aan het zingen. Toen glimlachte mijn moeder naar mij en mijn vader moedigde mij aan. Ik herinner me dat mijn hoofd recht boven de taart was en dat op een gegeven moment de kaarsjes uit waren. Iedereen juichte en klapte. Naderhand kwam één van mijn zussen naar mij toe en gaf me een knuffel en een kus. Ik herinner me dat het een blije en fijne verjaardag was. Ik ben nog steeds gek op die taart.

<u>Example 1 (English translation)</u>

I remember myself sitting at the end of a large table. On a chair next to my father, I think. The table was filled with sweets and they had my favorite cake, strawberry flavor and lots of cream. It had a little Mickey Mouse on top. Everyone from my closest family was there, my sisters, my grandparents, my aunts, uncles and cousins. We were sitting outside and the sun was shining. It must have been my birthday, because I remember candles being stuck in the middle of the cake. I think, I remember them to be blue. Everybody was singing. Then my mum smiled at me and my dad encouraged me. I remember that my head was right over the cake and that the candles were out at certain point. Everybody cheered and clapped. Afterwards one of my sisters came to hug me and gave me a kiss. I remember it to be a joyful and happy birthday. I still love that cake.

Voorbeeld 2

In mijn eerste herinnering neemt iemand mij op de fiets mee. Ik hou me ergens met mijn handen aan vast maar ik weet niet meer aan wat. Ik voel dat degene die fietst kracht zet om de pedalen rond te bewegen. Boem, boem, boem. Ik vind het moeilijk om rechtop in de wind te blijven zitten, mijn lijf wil alsmaar in elkaar duiken. Ik zie stoeptegels en het gras van een grasveld naast de stoep. Het was waarschijnlijk herfst of winter, of wanneer het ook maar vaak hard waait.

Example 2 (English translation)

My first memory is about someone taking me along on their bicycle. I am holding on to something with my hands but I don't know what. I feel that person who is riding the bicycle is using force to move the pedals around. Boom, boom, boom. I have difficulties sitting straight up in the wind, somehow my body wants to cower. I see the tiles of a pavement and the grass of a lawn next to it. I was probably autumn or winter, or whenever the winds are strong.

Voorbeeld 3

In mijn vroegste herinnering zat ik op de grond. Op een zo'n kleed waar een hele stad op getekend is, met straten en gebouwen. Mijn zus zat naast me. Ik denk dat we met Lego aan het spelen waren. Dat vonden we leuk toen we klein waren. Ik speelde eigenlijk best vaak met mijn zus als kind. Ik kan me niet echt herinneren wat er daarna gebeurde ... We vechten, maar ik weet niet zeker waarom. Ze slaat me met een stuk van de Lego. Ik huil .. ik word boos. Ik krab haar .. En toen kwam mijn moeder binnen. Dat is alles wat ik me herinner. Ik weet niet precies wat er verder gebeurde.

Example 3 (English translation)

In my earliest memory I was sitting on the floor. On one of those carpets that had an entire city drawn on it, with streets, and buildings. My sister was sitting next to me. I think we played with Lego. We loved to do that when we were younger. I actually played a lot with my sister when we were young. I can’t really remember what happened next… We fight, but I am not sure why. She hits me with a piece of Lego. I cry.. I get angry. I scratch her.. And then my mum came in. That’s all I remember. I don’t know exactly what happened next.
