# Supplementary material for: Manipulating the reported age in earliest memories in a Dutch community sample
Source: PLoS One. 2019 May 31;14(5):e0217436. doi: 10.1371/journal.pone.0217436 (PMC6544230; doi:10.1371/journal.pone.0217436)
Supplement: S3 File — (PDF) [file pone.0217436.s003.pdf]

S3 File

Rating scale used to assess memory characteristics

Rated on Likert scale:

Helemaal Oneens *Totally Disagree*					Niet eens of oneens *Neither agree nor disagree*					Helemaal eens *Totally Agree*
0	1	2	3	4	5	6	7	8	9	10
Scale										

Sensory details	(1)	Als ik me de ervaring herinner, kan ik dingen er van in mijn hoofd horen<br>*As I remember the event, I can hear it in my mind.*
	(2)	Als ik me de ervaring herinner, kan ik dingen er van voor mijn ogen zien<br>*As I remember the event, I can see things of it in front of my eyes.*
	(3)	Mijn herinnering bevat zintuiglijke informatie als geur, smaak of gevoel (gevoel zoals bij aanraken...)<br>*My memory contains sensory information such as smell, taste or feeling (feeling like touch)*
Vividness	(4)	Mijn herinnering is levendig<br>*My memory is vivid.*
	(5)	Mijn herinnering is gedetailleerd<br>*My memory is detailed.*
Place details	(6)	De plaats van de gebeurtenis is duidelijk.<br>*The location of the event is clear.*
	(7)	De omgeving in de herinnering is vertrouwd.<br>*The environment in the memory is familiar.*

Time perspective	(8)	De gebeurtenis lijkt lang te duren.<br>*The event seems to take a long time.*
Valence of memory	(9)	Over het algemeen is de herinnering positief.<br>*In general, the memory is positive.*
	(10)	Over het algemeen is de herinnering negatief *(reversed).*<br>*In general, the memory is negative.*
Memory of general feeling	(11)	Ik herinner me hoe ik me op het moment van de ervaring voelde.<br>*I remember how I felt at the moment of the experience.*
Valence of event	(12)	Toen ik de ervaring meemaakte waren mijn gevoelens positief.<br>*When I experienced the event, my feelings were positive.*
	(13)	Toen ik de ervaring meemaakte waren mijn gevoelens negatief *(reversed)*<br>*When I experienced the event, my feelings were negative.*
Emotional intensity of event	(14)	Toen ik de ervaring meemaakte waren mijn gevoelens sterk.<br>*When I experienced the event, my feelings were strong.*
Surrounding happenings	(15)	Ik herinner me dingen die onmiddellijk voor de gebeurtenis plaats hebben gevonden.<br>*I remember things that took place immediately <u>before</u> the event.*
	(16)	Ik herinner me dingen die onmiddellijk na de gebeurtenis plaats hebben gevonden.<br>*I remember things that took place immediately <u>after</u> the event.*
Sharing	(17)	Sinds deze gebeurtenis plaatsvond heb ik er vaak over nagedacht<br>*Since the event took place, I have often thought about it.*
	(18)	Sinds deze gebeurtenis plaatsvond heb ik er vaak over gesproken.<br>*Since the event took place, I have often talked about it.*
Visual perspective	(19)	In mijn herinnering zie ik door mijn eigen ogen wat er gebeurde.<br>*In my memory, I see through my own eyes what happened.*
	(20)	Ik zie de gebeurtenis alsof ik door de ogen van iemand anders kijk<br>*I see the event as if I am looking through someone else's eyes.*
Coherence	(21)	De volgorde van de gebeurtenissen in mijn herinnering is duidelijk<br>*The sequence of events in my memory is clear.*

Accessibility	(22)	Ik vond het makkelijk om op deze herinnering te komen.<br>*This memory was easy for me to recall.*
Control Question	(23)	De zon draait om de aarde.<br>*The sun revolves around the earth.*

6
