# Supplementary material for: Manipulating the reported age in earliest memories in a Dutch community sample
Source: PLoS One. 2019 May 31;14(5):e0217436. doi: 10.1371/journal.pone.0217436 (PMC6544230; doi:10.1371/journal.pone.0217436)
Supplement: S4 File — (PDF) [file pone.0217436.s004.pdf]

S4 File

Coding criteria memory types

Fragment memory: coded as 0

The fragmental memory is described as the earliest memory fragment of a person's life, it is a disconnected piece of memory. It lacks continuity, background information, and details associated with a story about an event. It might be remembered as an image, a mere sensory impression that might be visual, auditory, or of a smell, taste or touch, a behaviour, or an emotion. It is nothing more than an isolated fragment that sticks in someone's mind.

Features: isolated scene/percept, lack of context, no beginning or end, no reasoning (why's / because's); no temporal sequence

Example: My first memory is of the brightness of light—light all around. I was sitting among pillows on a quilt on the ground—very large white pillows. The quilt was a cotton patchwork of two different kinds of material—white with very small red stars spotted over it quite close together, and black with a red and white flower on it. I was probably eight or nine months old.

Snapshot memory: coded as 1

The Snapshot memory also refers to a memory fragment of a person's life, it is like a *mental picture* someone has in mind, a snapshot taken by the mind. This picture *may be very clear and detailed* just as a photo, but does *not tell a coherent story*. There may be background information provided which refer to that mental picture. There is no story-telling, thus there is also no

beginning or ending. The person simply describes the snapshot he/she has in mind. Consequently there is also no *coherent* temporal sequence of events happening. But it may include a sequence of several *disconnected* snapshots as long as it keeps its characteristics of a snapshot. In this case the person would report two or more distinct snapshots he/she has in his/her mind (e.g. snapshot 1 – blank – snapshot 2).

Features: isolated scene/percept, mental picture, context and details may be present; no beginning or end, no temporal sequence of events, no storyline

Example: I remember lying in a bed in my grandparent's house, in the small room on the left of the hallway. My grandfather was patting me on the back to help me get to sleep. A clock was ticking. My grandfather was wearing a white singlet.

Event memory: coded as 2

The event memory is a narrative story someone remembers. As in each story there should be a beginning and an end. The reported story usually contains a temporal sequence of events, thus words like then, before, after, etc are common. Details as well as background/context information may be provided, but do not have to be present as long as the memory is reported like a story. Oftentimes there is some sort of reasoning present (e.g. "we were eating cake, because it was my sisters birthday").

Features: a narrative structure: beginning, end, sequence of events (temporal order); context information, reasoning

Example: My brother and I head to clean our room. My parents really wanted us to do so, so they told us that they will come back after a while and everything which is on the floor then would land in the garbage can. My brother and I even asked if they promised to do so - they answered

yes and went out of the room. We took our garbage and spread it all over the floor. Everything we wanted to keep and all our toys were thrown on my brothers' bed. We were laughing all the time and when we finished we sat down on his bed between all the stuff and waited until our parents came back.

Repetitive: coded as 3

The repetitive memory refers to a memory of an *event that occurs/occured repetitively*, usually on a *regular basis* in a person's life. It might be a regular visit, or regular activity and should comprise of the same event or sequence of events. A category of events.

Example: Every Sunday we went to church with the whole family.

General/autobiographical memory or association/autobiographical fact: coded as 4

- General: Broad period of one's life

  Example: my childhood was happy
- Associations refer to a single word or a few words being connected to the childhood.

  Example: Klasse, leuke tijd gehad, kleuterschool
- A*utobiographical fact* simply refers to a fact about a person. It might concern that person's living arrangements, home country or family situation.

  Example: We were living in Los Angeles.

Not to be coded: coded as 9

In case of blanks the participant might have a memory in mind but did not want to (and also did not) report such memory. Other variables need be considered (such as estimation of age or strategy used) in the analyses
