# Supplementary material for: Manipulating the reported age in earliest memories in a Dutch community sample
Source: PLoS One. 2019 May 31;14(5):e0217436. doi: 10.1371/journal.pone.0217436 (PMC6544230; doi:10.1371/journal.pone.0217436)
Supplement: S1 Table — (PDF) [file pone.0217436.s005.pdf]

S1 Table

Distribution of participants across strata

Table giving a detailed overview of how many participants remained in each of the strata and how instruction conditions were distributed across strata

Age	Gender	Education Level	Condition			
			**Early (n = 203)**	**Late (n = 203)**	**Control (n = 212)**	**Total**
20-29	Men	High	8	10	6	24
		Middle	8	8	8	24
		Low	8	7	8	23
		Total Men	24	25	22	71
	Women	High	11	10	10	31
		Middle	8	10	9	27
		Low	7	7	8	22
		Total Women	26	27	27	80
		**TOTAL**	**50**	**52**	**49**	**151**
30-39	Men	High	8	9	8	25
		Middle	5	4	9	18
		Low	7	4	7	18
		Total Men	20	17	24	61
	Women	High	10	8	9	27
		Middle	10	10	10	30
		Low	8	9	8	25
		Total Women	28	27	27	82
		**TOTAL**	**48**	**44**	**51**	**143**
40-49	Men	High	10	10	9	29
		Middle	8	9	8	25
		Low	7	9	7	23
		Total Men	25	28	24	77
	Women	High	9	10	10	29
		Middle	9	10	10	29
		Low	9	9	9	27
		Total Women	27	29	29	85
		**TOTAL**	**52**	**57**	**53**	**162**
50-59	Men	High	10	9	8	27
		Middle	10	9	11	30
		Low	6	7	9	22

	Total Men	26	25	28	79
Women	High	10	9	13	32
	Middle	10	8	8	26
	Low	8	8	10	26
	Total Women	28	25	31	84
	**TOTAL**	**53**	**50**	**59**	**163**
