# Supplementary material for: Manipulating the reported age in earliest memories in a Dutch community sample
Source: PLoS One. 2019 May 31;14(5):e0217436. doi: 10.1371/journal.pone.0217436 (PMC6544230; doi:10.1371/journal.pone.0217436)
Supplement: S2 Table — (PDF) [file pone.0217436.s006.pdf]

S2 Table

Tables of ages estimates per strata

Table of Means (SDs) and Cell Sizes per Age-Group Gender, and Educational Level across the Early, Late and Control Conditions.

		Early		Late		Control	
		n	*M (SD)*	*n*	*M (SD)*	*n*	*M (SD)*
**Age**							
	20-29	50	44.74 (19.13)	52	55.77 (27.11)	49	52.55 (22.46)
	30-39	48	43.44 (20.51)	44	58.32 (22.32)	51	55.71 (24.03)
	40-49	52	49.69 (20.19)	57	52.65 (52.65)	53	51.70 (23.96)
	50-59	54	52.22 (26.08)	50	57.94 (57.94)	59	59.75 (22.00)
**Gender**							
	Male	95	51.07 (21.24)	95	53.94 (25.43)	98	57.35 (25.43)
	Female	109	44.72 (22.08)	108	57.78 (25.42)	114	53.17 (20.94)
**Educational Level**							
	High	76	45.46 (21.35)	75	54.59 (24.85)	73	52.64 (22.26)
	Middle	68	45.10 (22.26)	68	53.18 (23.52)	73	53.74 (23.98)
	Low	60	53.40 (21.38)	60	60.90 (27.86)	66	59.32 (23.00)

**Grand Mean**	**204**	**47.68 (21.88)**	**203**	**55.98 (25.43)**	**212**	**55.10 (23.17)**

Table of Means (SDs) for Age-Group (20-29) per Educational Level (High / High + Middle) across the Early, Late and Control Conditions

		**Early**		**Late**		**Control**	
		n	*M (SD)*	*n*	*M (SD)*	*n*	*M (SD)*
**Age** (20-29)							
	High	19	46.63 (10.63)	20	61.90 (29.92)	16	49.38 (13.61)
	Middle	16	46.44 (23.65)	18	49.00 (18.28)	17	52.59 (27.38)
	Low	15	40.53 (12.63)	14	55.71 (31.87)	16	55.69 (24.65)

Note. The middle education group within this young age group contains participants who would be eligible to study at academic or applied universities, but would be too young to have finished that higher education. Therefore, they would not be categorized in the high education group. However, the combination of the high and middle education level groups would be the closest approximation of the typical undergraduate sample in most studies in psychology.
